# NOVEL NUTRITION EQUITY METHOD: OLDER ADULT NUTRITION EQUITY INDEX (NEI) DEVELOPED IN HEALTH ABC & EXPANDING TO PHRESH

**DOI:** 10.1093/geroni/igae098.4031

**Published:** 2024-12-31

**Authors:** Aarohee Fulay, Tamara Dubowitz, Samaneh Farsijani, Kerri Freeland, Jimmie Roberts, Andrea Rosso, Denise Houston, Elsa Strotmeyer

**Affiliations:** University of Pittsburgh School of Public Health, Pittsburgh, Pennsylvania, United States; University of Pittsburgh School of Public Health, Pittsburgh, Pennsylvania, United States; University of Pittsburgh, Pittsburgh, Pennsylvania, United States; University of Pittsburgh, Pittsburgh, Pennsylvania, United States; Hebrew SeniorLife, Boston, Massachusetts, United States; University of Pittsburgh, Pittsburgh, Pennsylvania, United States; Wake Forest University School of Medicine, Winston Salem, North Carolina, United States; University of Pittsburgh, Pittsburgh, Pennsylvania, United States

## Abstract

Nutrition equity – no barriers to obtaining healthy foods – is critical for older adults. Current nutrition equity research lacks sufficient attention to multiple and concurrent processes that may contribute to acquiring healthy food. For example, improving food security, i.e. fiscal ability to obtain healthy foods, alone may not improve diet without accounting for geospatial food access or individual physical limitations. We designed a novel nutrition equity index (NEI) for older adults which combines food security, food access, and physical limitations data from older Black and White adults in Pittsburgh, PA and Memphis, TN from the Health, Aging, and Body Composition Study (Health ABC; 51.7% women; 37.9% Black; 74.7±2.9 years). In multivariable linear regression, low (12.1%) and moderate nutrition equity (32.5%) vs. high were associated with lower dietary quality (measured via Healthy Eating Index), though after adjusting for race (which was included as a marker for social/structural factors), this NEI association attenuated to non-significance, suggesting effect modification. We will extend our analysis to examine nutrition equity with a historically marginalized population living in two predominantly Black neighborhoods by using recent data from Pittsburgh Hill/Homewood Research on Neighborhood Change and Health (PHRESH; 80.7% women; 94.1% Black; 64.8±9.3 years). We will expand the NEI to include additional food access (e.g. distance to major food shopping venue) and functional ability measures in an expanded population of middle-aged to older adults. Expansion of the NEI and its application in this new population/setting will highlight the structural nutrition equity components which are critical to address.

